# The Role of hBRCA2 in the Repair of Spontaneous and UV DNA Damage in *Saccharomyces cerevisiae*

**DOI:** 10.17912/micropub.biology.001161

**Published:** 2024-04-18

**Authors:** Sherrice Law, David Park, Hannah Park, Hannah Zhang, Damon Meyer

**Affiliations:** 1 College of Medicine, California Northstate University, Elk Grove, California, United States; 2 College of Health Sciences, California Northstate University, Elk Grove, California, United States

## Abstract

Women with mutations in the human BRCA2 gene ( *hBRCA2* ) have an increased risk of developing breast and ovarian cancer throughout their lifetime. *hBRCA2* transcribes proteins necessary for gene repair through homologous recombination (HR). In order to better understand the role of hBRCA2 in response to specific types of DNA damage, the present study evaluated HR in the budding yeast, *Saccharomyces cerevisiae* , using wildtype (WT) and *rad52Δ* mutant cells subject to spontaneous and UV damage in the presence or absence of hBRCA2. As expected, *rad52Δ* genotypes yielded lower recombination frequencies compared to WT in both spontaneous and UV exposure experiments. However, there was no significant difference between *rad52Δ* mutants with or without hBRCA2. Interestingly, higher UV exposure resulted in a relative increase in HR for only the *rad52Δ* mutant genotypes. The results demonstrate that hBRCA2 complementation may not be as substantial in spontaneous or UV DNA damage compared to double-strand break DNA damage, as previous work has shown.

**Figure 1. Recombination frequencies of Diploid WT and rad52Δ genotypes with and without hBRCA expression following spontaneous or UV damage:**
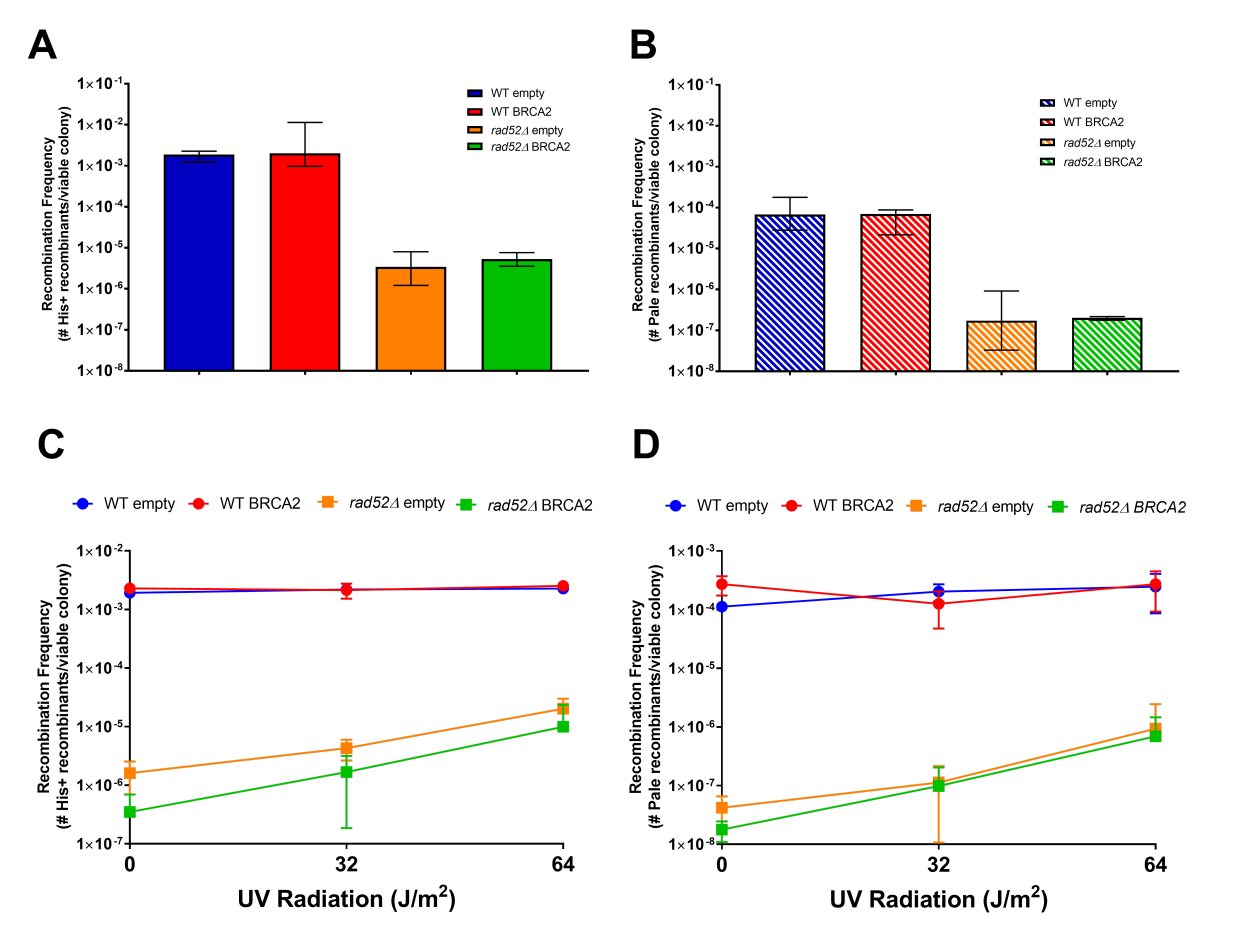
**(A)** His+ recombinant or **(B)** pale recombinant frequencies of diploid WT and *rad52Δ* mutants with empty or hBRCA2 expressing plasmids after spontaneous DNA damage. Median values and 95% CI were calculated from a minimum of ten independent cultures. **(C)** His+ recombinant or **(D)** pale recombinant frequencies of diploid WT and *rad52Δ* mutants with empty or hBRCA2 expressing plasmid after UV-radiation exposure of 0, 32, or 64 J/m ^2^ . Mean values and standard error mean were calculated from a minimum of three independent cultures.

## Description

*BRCA2* is one of the two human tumor suppressor genes that encode proteins facilitating the pairing of homologous DNA strands and strand exchange during HR repair (Jensen et al., 2010, Le at al., 2021) . Dysfunction in this critical gene can lead to breast and ovarian cancer in humans (Petrucelli et al., 2010) . Yeast has been utilized as a model to study human BRCA2 (hBRCA2) influence on cell metabolism, programmed cell death, and homologous recombination, which provides insight into the function of hBRCA2 in mammalian cells (Spugnesi et al., 2013; Guaragnella et al., 2014a; Guaragnella et al., 2014b; Costanza et al., 2022; Lodovichi et al., 2022) . Previous work showed that hBRCA2 can partially complement the loss of yeast Rad52 in homologous recombination initiated by an induced double-strand break, suggesting functional overlap between hBRCA2 and yeast Rad52 (Law et al., 2023) . While Spugnesi et al. (2013) showed hBRCA2 expression influences MMS resistance, the extent to which hBRCA2 complementation impacts the response to other forms of DNA damage remains unclear. One explanation for hBRCA2 complementation of Rad52 may be due, in part, to the bipartite binding of Rad51 observed in both proteins (Deveryshetty et al., 2023) and the observed interaction between hBRAC2 and yeast Rad51 (Jensen et al., 2010) . Thus, the current study aimed to investigate the role of hBRCA2 in the HR repair of spontaneous and UV-induced DNA damage within *rad52Δ* mutant yeast cells. It was hypothesized that hBRCA2 would be able to complement the lowered HR frequency in *rad52Δ* mutants following spontaneous DNA damage but may not be able to complement UV-induced DNA damage.

To carry out our study, HR was assessed in diploid WT and *rad52Δ* mutant cells by analyzing recombinants between two truncated *his3* alleles ( *his3Δ3’* and *his3Δ5’* ) on homologous chromosomes after spontaneous or UV-induced DNA damage. Specifically, HR between the *his3Δ3’* and *his3Δ5’* alleles can generate a functional *HIS3* gene which allows the cells to grow on medium lacking histidine and are referred to as His+ recombinants. Alternatively, pale recombinants can be created when the *his3Δ3’* and *his3Δ5’* alleles recombine to create *his3Δ5’* homozygotes (Law et al., 2023) . The expression of hBRCA2 via western blot was shown previously in identical yeast strains that are used in the current analysis (Law et al., 2023) . WT and *rad52Δ* mutant cultures possessing both *his3Δ3’* and *his3Δ5’* alleles with or without a hBRCA2 expressing plasmid were plated onto YPD and medium lacking histidine (SD-His) using appropriate dilutions. Pale colonies on YPD were examined to note the number of pale recombinants, while colonies growing on SD-His were scored as His+ recombinants. The His+ recombinant frequencies were calculated from the number of colonies on SD-His relative to the total number of viable cells, as determined by the number of YPD colonies. The pale recombinant frequencies were calculated from the number of pale colonies on YPD to the total number of colonies on YPD.

Mutant *rad52Δ* genotypes yielded lower His+ and pale recombination frequencies compared to WT in both spontaneous and UV exposure experiments ( Figure 1, A -D). This result supports the established finding that Rad52 plays a critical role in DNA repair caused by spontaneous and UV-induced DNA damage (Coic et al., 2008). Importantly, there was no significant difference between *rad52Δ* mutants with or without hBRCA2, demonstrating that hBRCA2 did not complement the loss of Rad52 in spontaneous and UV-induced DNA damage repair ( Figure 1, A -D). This suggests the mechanism of DNA repair in generating His+ and pale recombinants in response to spontaneous and UV-induced damage is a unique process requiring yeast Rad52 that can not be replaced by hBRCA2. In addition, our results support hBRCA2 not being directly involved in spontaneous or UV-mediated DNA damage repair as shown previously (Patel et al., 1998) , but is the consequence of down-regulation of expression following UV exposure (Wang et al., 2001) . The above results are in contrast to previous findings that demonstrated hBRCA2 partially complemented *rad52Δ* mutants in the repair of induced DSBs (Law et al., 2023) .

Interestingly, higher UV exposure resulted in a relative increase in HR for only the *rad52Δ* mutant genotypes, providing evidence for a Rad52-independent process producing recombinants similar to results previously published (Haber & Hearn, 1985; Coic et al., 2008) . Although it is unclear if the Rad52-independent mechanism we observed relies on Rad50 and Rad51 as shown by others (Coic et al., 2008), our findings demonstrate that this process is not as efficient as Rad52-dependent repair and thus is masked in the presence of a functioning Rad52. Consequently, when Rad52 is knocked out, the yeast will resort to the suboptimal pathway for UV-damage repair to produce recombinants. The results of our study build upon previous work which supports utilizing yeast as a model organism to study the functionality of hBRCA2 and continue exploring its role in HR repair. Specifically, continued analysis of known and unknown hBRCA2 variants in yeast will help contribute to the current understanding of breast and ovarian cancer research (Spugnesi et al., 2013; Lodovichi et al., 2022) .

## Methods

**Strains and Plasmids**

WT and *rad52Δ* mutant *S. cerevisiae* genotypes and hBRCA2 expression plasmid were provided by the Heyer lab and previously described (Law et al., 2023) . All yeast strain are derived from a W303 background.

**Recombination Assay**

The DSB repair assay used was described previously (Law et al, 2023) and utilized two truncated *his3* alleles ( *his3Δ3’* and *his3Δ5’* ) to measure recombination frequency. Single colonies possessing the appropriate genotype were used to inoculate two mL of SD-URA medium (0.67% (wt/vol) yeast nitrogen base w/out amino acids, amino acid dropout mix w/out uracil, and 2% (wt/vol) glucose). The inoculated cultures were incubated for 48 hrs at 30°C before plating onto YPD (1% (wt/vol) yeast extract, 2% (wt/vol) peptone, 2% (wt/vol) glucose) and medium lacking histidine (SD-His) using appropriate dilutions. Cells not exposed to UV were placed at 30°C directly after plating for five days before counting. To determine the effect of UV radiation on repair frequencies, the plated cells were irradiated with 32, or 64 J/m ^2^ of UV and then incubated at 30°C for five days before counting. The HR frequency was determined by dividing the number of histidine prototrophic colonies by the total number of viable cells as determined by plating dilutions onto YPD. In addition, pale colonies were counted from the YPD media and divided by the total number of colonies on YPD.
